# Electronic–Oxygen Synergy at Ca-Fe Dual-Metal Interfaces for Selective Syngas Regulation in Biomass Chemical Looping Gasification

**DOI:** 10.3390/molecules30071471

**Published:** 2025-03-26

**Authors:** Yijie Wang, Jiajie Li, Sitao Zhu, Michael Hitch

**Affiliations:** 1 School of Energy and Environmental Engineering, University of Science and Technology Beijing, Beijing 100083, China; wangyijie_ustb@126.com; 2 Beijing Key Laboratory of Resource-Oriented Treatment of Industrial Pollutants, University of Science and Technology Beijing, Beijing 100083, China; 3 School of Resource and Safety Engineering, University of Science and Technology Beijing, Beijing 100083, China; 4 Key Laboratory of Efficient Mining and Safety of Metal Mines, Ministry of Education, University of Science and Technology Beijing, Beijing 100083, China; 5 Faculty of Science, University of the Fraser Valley, Abbotsford, BC V2S 7M8, Canada; michael.hitch@ufv.ca

**Keywords:** chemical looping gasification, Ca_2_Fe_2_O_5_ oxygen carrier, biomass conversion, syngas production, DFT calculations

## Abstract

This study reveals the efficient catalytic role of Ca-Fe-based oxygen carriers (Ca_2_Fe_2_O_5_) in biomass chemical looping gasification. With oxygen carrier introduction, the CO yield doubled (0.13 Nm^3^/kg→0.26 Nm^3^/kg), with 76.10% selectivity. Steam co-feeding further increased the H_2_ yield from 0.19 Nm^3^/kg to 0.72 Nm^3^/kg, significantly elevating the H_2_/CO ratio to 2.62. Combined with density functional theory (DFT), the micro-mechanism of reduced oxygen carrier surfaces activating CO_2_/H_2_O was elucidated. CO_2_ (adsorption charge −0.952 |e|) and H_2_O (adsorption charge −0.612 |e|) chemically adsorb at the CaO(111)/Fe(110) interface, where Fe atoms (charges 0.433 |e|, 0.927 |e|) act as electron donors to drive efficient molecule activation. CO_2_ undergoes single-step splitting (CO_2_→CO* + O*), with the desorption energy barrier (*E*_a_ = 1.09 eV, 105.17 kJ/mol) determining the reaction rate. H_2_O splits via two-step cleavage (H_2_O→HO* + H*→2H* + O*), which is rate-limited by the first step (*E*_a_ = 0.42 eV, 40.52 kJ/mol). Simultaneously, the reduced oxygen carrier achieves oxidative regeneration through surface O* lattice incorporation. This work atomically reveals the “electron transfer–oxygen transport” synergy at the Ca-Fe bimetallic interface, establishing a theoretical framework for the directional regulation of the syngas composition and the design of high-performance oxygen carriers.

## 1. Introduction

The urgent need for global energy structure transformation has driven the rapid development of renewable energy technologies represented by biomass energy. As a carbon-neutral renewable energy source that can be directly converted into high-grade fuels, the efficient utilization of biomass energy holds strategic significance in achieving “dual-carbon” goals [1,2,3]. According to International Energy Agency (IEA) statistics, the global annual utilizable biomass resources exceed 1.3 × 10^11^ tons of standard coal equivalents, but their energy conversion efficiency remains below 10% [4]. High-efficiency biomass gasification technology has become one of the focal points in international energy research under carbon neutrality objectives [5,6]. However, traditional gasification processes face bottlenecks, such as the insufficient secondary cracking of tar, low product selectivity (uncontrollable H_2_/CO ratio) and inadequate carbon conversion rates [7,8,9]. These limitations reduce the system efficiency and increase the processing costs, severely constraining large-scale applications.

In recent years, chemical looping gasification technology has achieved the directional conversion of biomass into syngas (H_2_/CO) through redox cycles of oxygen carriers [10,11,12]. A typical chemical looping gasification system consists of a fuel and air reactor, where the former facilitates biomass gasification. At the same time, the latter enables the combustion of incompletely converted biomass and oxygen carrier regeneration [12]. In chemical looping gasification systems, oxygen carriers (typically metal oxides like Fe_2_O_3_) utilize lattice oxygen instead of O_2_ as the oxygen source for fuel reactors [13,14], thereby eliminating the oxygen preparation costs [15]. The incomplete oxidation of biomass with oxygen carriers produces CO, enhancing the gas’ calorific value. Metal oxide oxygen carriers also exhibit catalytic effects on biomass tar cracking [16,17,18], addressing traditional gasification’s shortcomings.

**Figure 1:**
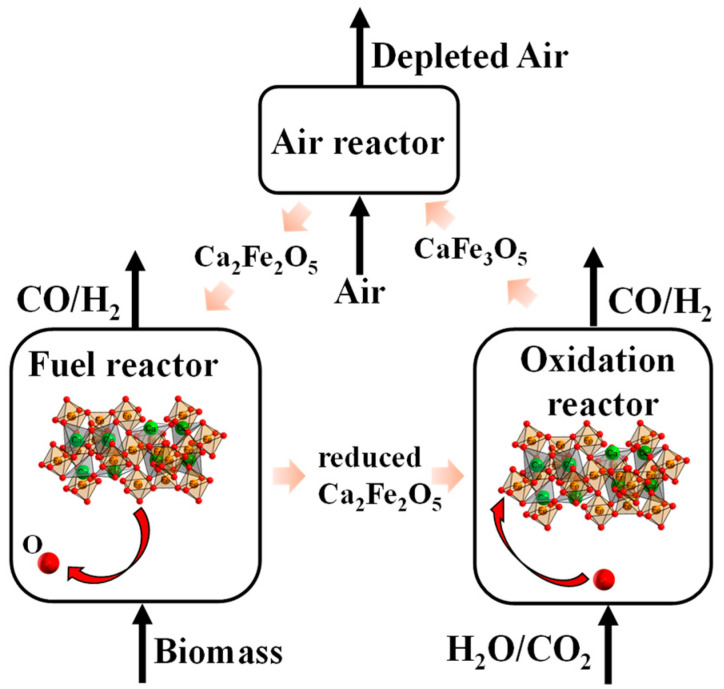
Schematic of chemical looping gasification–water splitting and chemical looping gasification–CO_2_ splitting.

Building upon chemical looping gasification, recent advancements include chemical looping gasification–water splitting (CLGWS) and chemical looping gasification–CO_2_ splitting (CLGCS) technologies [19,20,21]. These systems integrate splitting processes to convert CO_2_/H_2_O into CO/H_2_, as illustrated in Figure 1. By introducing H_2_O and CO_2_ between the fuel and air reactors, the reduced oxygen carriers undergo oxidation to generate high-purity H_2_ and CO. Subsequently, the partially oxidized oxygen carrier is completely regenerated in air reactors. Both chemical looping gasification–water splitting and chemical looping gasification–CO_2_ splitting processes rely critically on the oxygen carrier performance [22,23,24,25], which directly determines the gas product quality and yield [26].

Recent studies reveal that composite oxygen carriers [27] can overcome the limitations of single-metal oxygen carriers, such as their poor cycling capacity, instability and low reactivity [28]. Ca-based oxygen carriers have gained attention in chemical looping gasification research due to their cost-effectiveness, ability to reduce the CO_2_ yield and tar catalytic cracking properties [28,29,30,31,32]. Chan et al. [33] demonstrated that CaO/Fe_2_O_3_ oxygen carriers can enhance syngas production with high H_2_/CO ratios through the co-gasification of biomass and polyethylene.

Performance analyses of Ca-Fe composite oxygen carriers show that Ca_2_Fe_2_O_5_ significantly improves the steam conversion efficiency and H_2_ yield during gasification [34]. Its moderate oxidation capacity facilitates high-quality syngas production [35].

Density functional theory (DFT) calculations have proven effective in investigating the geometric and electronic structures of metal oxide interfaces, providing atomic-level insights into their properties [36,37,38,39,40]. Feng et al. [37] employed DFT to explore the CH_4_ conversion pathways in Ca_2_Fe_2_O_5_ oxygen carriers, offering theoretical support for enhanced CH_4_ conversion efficiency. DFT revealed that [39] Ni doping lowers the oxygen vacancy formation energy and enhances CO_2_ adsorption, promoting CO_2_ activation and splitting. Thus, DFT has enabled novel chemical looping gasification systems for CO_2_ utilization.

This study investigates the direct/steam chemical looping gasification of Ca_2_Fe_2_O_5_ oxygen carriers with *Chlorella vulgaris* (biomass) through comparative experiments with blank (SiO_2_) and steam groups, analyzing the oxygen carrier regulation mechanisms through carbon conversion, H_2_/CO selectivity and the gas calorific value. Using DFT simulations, we construct a CaO(111)/Fe(110) composite interface model to elucidate the complete adsorption–activation–dissociation process of CO_2_/H_2_O molecules on reduced Ca_2_Fe_2_O_5_ oxygen carriers (dual-metal sites), including the adsorption energy, transition state geometries and electronic density of state variations. The findings can guide the development of highly stable and selective oxygen carriers in chemical looping gasification–water splitting and chemical looping gasification–CO_2_ splitting processes.

## 2. Results and Discussion

### 2.1. Chemical Looping Gasification Experimental Results

**Figure 2:**
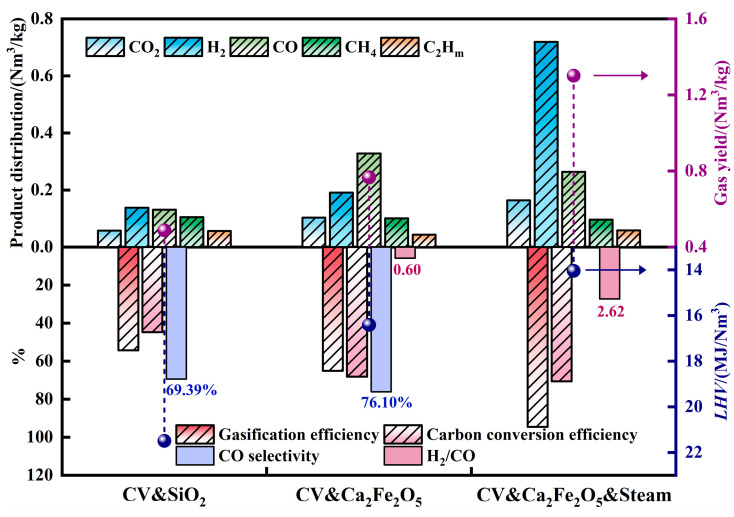
Gas production results in different conditions.

The experimental conditions for chemical looping gasification are listed in Table 1, labeled as A1, A2 and A3. The chemical looping gasification gas production results for each group are shown in Figure 2.

Compared to A1 (without the oxygen carrier), A2 exhibited significant increases in the carbon conversion rate and total gas yield, indicating that adding the oxygen carrier promoted biomass gasification. Although the lower heating value (LHV) of the gas of A2 decreased due to reduced concentrations of high-calorific components such as CH_4_ and C_2_H_m_, the yields of CO_2_, H_2_ and CO all showed positive growth. Notably, the CO gas yield remarkably increased from 0.13 Nm^3^/kg to 0.26 Nm^3^/kg, doubling in value. The CO selectivity of A2 improved to 76.10% relative to A1, demonstrating that CO_2_ was selectively converted to CO under the influence of the oxygen carriers. Previous studies have confirmed that reduced-state Ca_2_Fe_2_O_5_ can decompose CO_2_ into CO [21].

When steam was introduced to A2 (forming A3), the carbon conversion rate and total gas yield continued to rise. The H_2_ gas yield increased substantially from 0.19 Nm^3^/kg to 0.72 Nm^3^/kg, and the H_2_/CO ratio significantly improved from 0.60 to 2.62. This indicates that oxygen carriers can effectively enhance H_2_ production. Research by [20,35,39] revealed that reduced Ca_2_Fe_2_O_5_ undergoes steam oxidation regeneration to its initial state while efficiently releasing H_2_.

To further investigate the microscopic interaction mechanisms between reduced Ca_2_Fe_2_O_5_ and H_2_O/CO_2_, this study employed DFT to calculate and simulate energy exchange and reaction pathways. Before the calculations, the reduced Ca_2_Fe_2_O_5_ was characterized to ensure the reliability of the model construction.

### 2.2. Characterization of Oxygen Carriers

**Figure 3:**
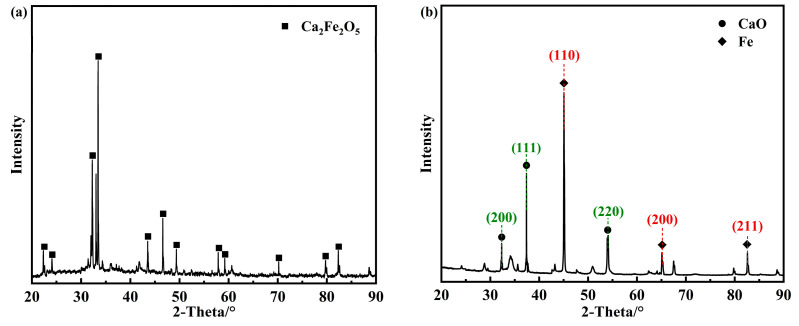

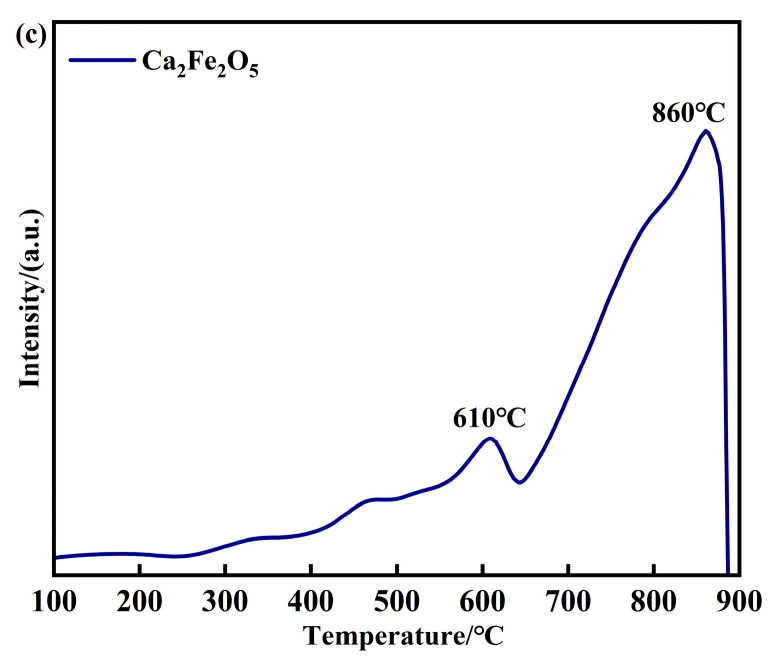
(**a**) XRD patterns of fresh Ca_2_Fe_2_O_5_; (**b**) XRD patterns of H_2_-reduced Ca_2_Fe_2_O_5_; (**c**) H_2_-TPR profiles of Ca_2_Fe_2_O_5_.

The physicochemical properties of the Ca_2_Fe_2_O_5_ oxygen carrier before and after reduction were analyzed by XRD, as shown in Figure 3a,b. The reduction performance of Ca_2_Fe_2_O_5_ was investigated through H_2_-TPR testing, with the results shown in Figure 3c.

As shown in Figure 3a, the primary component of the fresh oxygen carrier was Ca_2_Fe_2_O_5_. Figure 3c reveals two characteristic reduction peaks for Ca_2_Fe_2_O_5_ at 610 °C and 860 °C. Combined with the phase analysis of the reduced sample by XRD (Figure 3b), the high-intensity characteristic peaks at 37.3° and 44.8° correspond to the (111) crystal plane of CaO and the (110) crystal plane of Fe, respectively. It is inferred that these two reduction peaks correspond to the two-step reduction process: Ca_2_Fe_2_O_5_→FeO + CaO and FeO→Fe. These characterization results indicate that Ca_2_Fe_2_O_5_ preferentially exposes the CaO(111)/Fe(110) crystal planes during reduction. The CaO(111)/Fe(110) composite interface was selected as the simplified computational model for reduced Ca_2_Fe_2_O_5_ to balance computational efficiency and model representativeness.

### 2.3. CO_2_ Adsorption and Splitting

Based on literature reports and the gasification experiments described in Section 2.1, the reaction between CO_2_ (as a gaseous oxidizer) and the reduced oxygen carrier is the key step for CO generation. This study employs DFT simulations to investigate the CaO(111)/Fe(110) composite interface system, aiming to reveal the dynamic activation mechanism of CO_2_ molecules at the interface.

**Figure 4:**
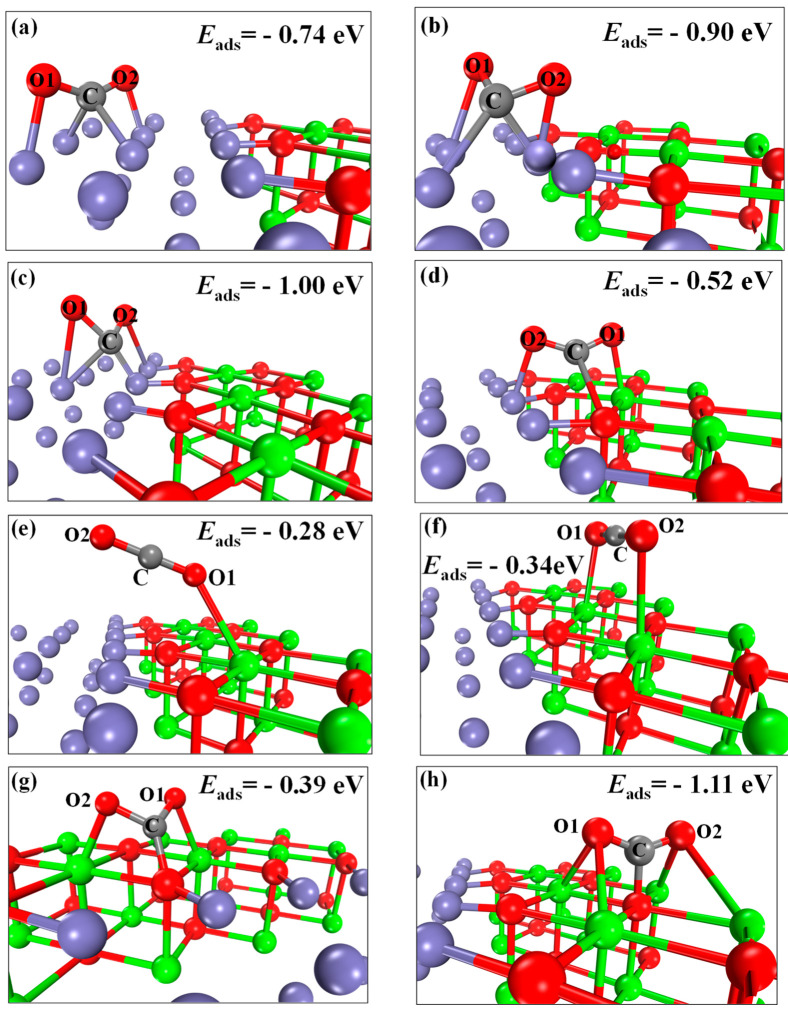
Adsorption configurations of CO_2_ on the CaO(111)/Fe(110) surface: (**a**–**c**) Fe-Fe bridge; (**d**) O-top; (**e**) Ca-top; (**f**) Ca-Ca bridge; (**g,h**) O-top.

**Figure 5:**
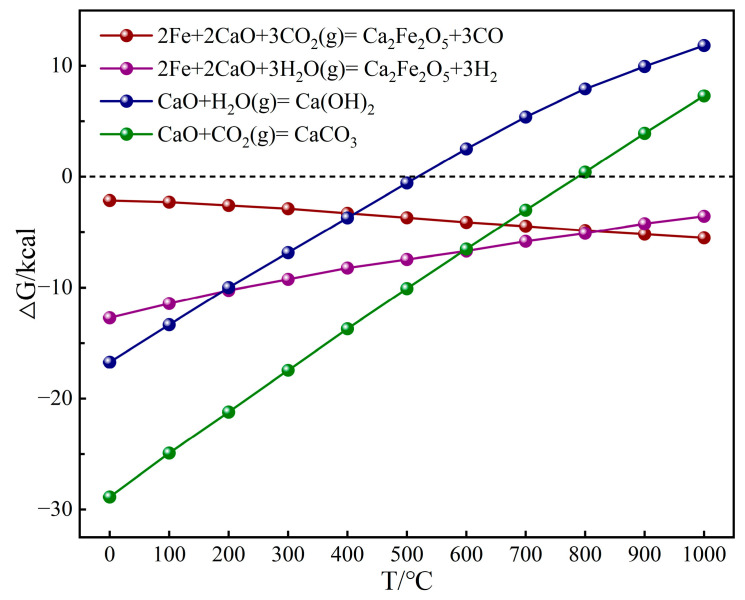
△G of different reactions.

The adsorption configurations of CO_2_ on the CaO(111)/Fe(110) composite interface are shown in Figure 4a–h. Configurations (a)~(c) represent three adsorption configurations of CO_2_ at Fe-Fe bridge sites. Configuration (d) corresponds to CO_2_ adsorption at the O top site between Ca and Fe. Configuration (e) shows CO_2_ adsorption at the Ca top site. Configuration (f) demonstrates CO_2_ adsorption at the Ca-Ca bridge site. Configurations (g) and (h) illustrate CO_2_ adsorption at the O top sites between Ca and Ca. The adsorption energies for (a)~(h) are −0.74, −0.90, −1.00, −0.52, −0.28, −0.34, −0.39, and −1.11 eV (−71.40, −86.84, −96.49, −50.17, −27.02, −32.81, −37.63, and −107.10 kJ/mol), respectively. A more negative adsorption energy (larger absolute value) indicates lower system energy and greater structural stability. Thus, configuration (c) is the most stable for CO_2_ adsorption on Fe sites, and configuration (h) is the most stable on CaO sites. Configuration (h) corresponds to CO_2_ chemisorption at alkaline CaO sites, forming CaCO_3_. However, as shown in Figure 5, at 800 °C, the standard Gibbs free energy △G > 0 of CaCO_3_, making it thermodynamically unstable. In contrast, the reaction between Fe atoms on CaO(111)/Fe(110) and CO_2_ exhibits a negative △G (△G < 0), indicating that the reaction can proceed spontaneously. Consequently, configuration (c) becomes the optimal stable state. In this configuration, the C-O1 and C-O2 bond lengths measure 1.2863 Å and 1.2990 Å, respectively, exceeding the original C-O bond length of CO_2_ (1.1768 Å) by 0.1095 Å and 0.1222 Å. This elongation unequivocally confirms CO_2_ activation at Fe catalytic sites.

**Figure 6:**
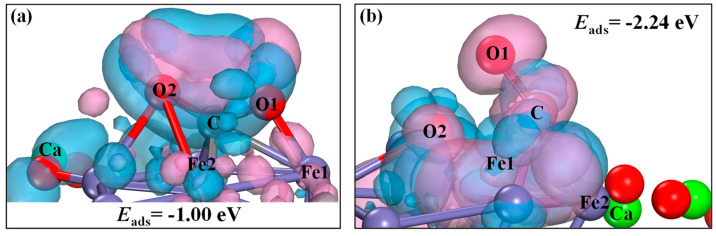
Optimized structures and deformation charge density of CO_2_ adsorption over CaO(111)/Fe(110) surface: (**a**) configuration a; (**b**) configuration b.

Figure 6 presents the geometry and charge redistribution analysis for CO_2_ adsorption on CaO(111)/Fe(110). Figure 6a represents the most stable configuration (c) from Figure 4, where CO_2_ chemisorbs atop an Fe atom, forming an Fe-C bond (1.9218 Å) with an adsorption energy of −1.00 eV (−96.49 kJ/mol). In the deformation charge density map, blue regions denote electron gains (acceptors), and pink regions denote electron losses (donors). The analysis reveals charge transfer during adsorption: C, O1 and O2 act as electron acceptors, while Fe1 and Fe2 act as electron donors. This indicates electron transfer from reduced Ca_2_Fe_2_O_5_ to adsorbed CO_2_. A Bader charge analysis (Table 2) shows that CO_2_ in configuration (a) gains −0.952 |e|, acting as an electron acceptor. In Figure 6b, configuration (b) represents CO_2_* (after electron gain) forming CO* via O migration. Configuration (b) has an adsorption energy of −2.24 eV (−216.13 kJ/mol), indicating higher stability. The deformation charge density analysis shows C, O2 and Fe1 as electron acceptors and O1 and Fe2 as electron donors. The total charges of CO_2_* before and after adsorption are −0.963 |e| and −1.421 |e|, respectively, confirming CO* as an electron acceptor.

**Figure 7:**
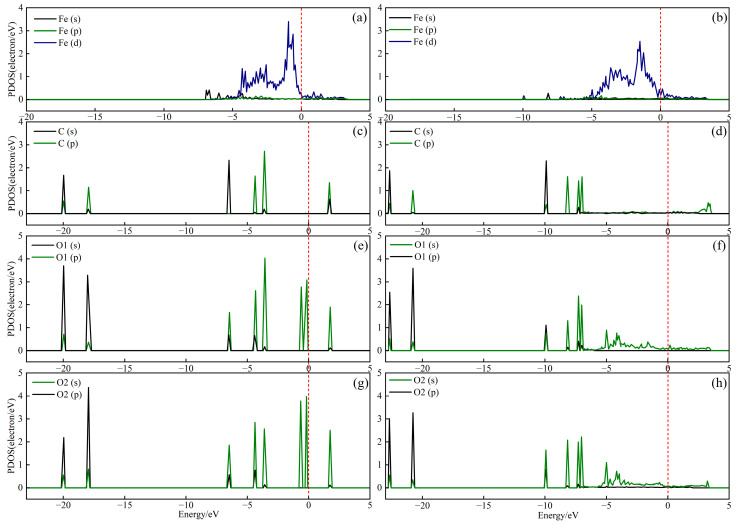
PDOS of CO_2_ adsorption on CaO(111)/Fe(110) surface: (**a**,**c**,**e**,**g**) before adsorption; (**b**,**d**,**f**,**h**) after adsorption.

To elucidate the electronic interactions between CaO(111)/Fe(110) and CO_2_, the partial density of states (PDOS) of C, O and Fe atoms before and after CO_2_ adsorption on CaO(111)/Fe(110) was calculated. The PDOS reveals the electronic structure and chemical bonding characteristics by decomposing the density of states into individual atomic orbitals (e.g., s, p and d orbitals). As shown in Figure 7, the bonding strength between C and O atoms is reflected by the overlapping of the peaks and their positions relative to the Fermi level (0 eV). Comparing the PDOS results before and after adsorption (Figure 7a,b), new peaks emerge at −8.16 eV and −9.93 eV, indicating the formation of new chemical bonds between Fe atoms and the C atom of CO_2_ after adsorption. Figure 7b,d demonstrate significant peak overlap between C and Fe atoms at −8.16 eV and −9.93 eV post-adsorption. Comparing Figure 7e,f, as well as Figure 7g,h, new peaks at the same positions as in Figure 7b appear in Figure 7f,h. This suggests that interactions between Fe atoms and the O atoms of CO_2_ create lower-energy orbitals, weakening the covalent bond between C and O. In Figure 7f,h, the p orbitals of O atoms near the Fermi level shift to lower-energy states. Similarly, the d orbitals of Fe in Figure 7b and the p orbitals of C in Figure 7d shift to lower energy levels. These observations imply that interactions between CO_2_ and Fe atoms in CaO(111)/Fe(110) reduce the system’s energy, leading to the formation of new molecular orbitals.

**Figure 8:**
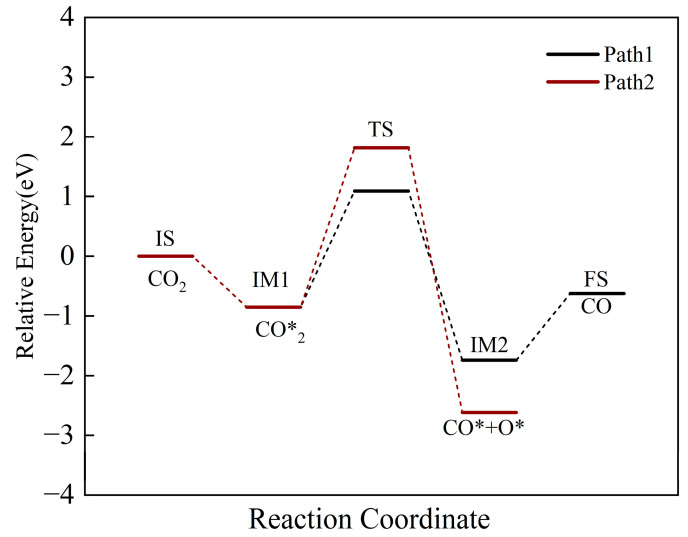
The energy profile of intermediates, transition states and final states of CO_2_ splitting on the CaO(111)/Fe(110) surface.

Starting from the most stable adsorption configuration, the reaction pathways of CO_2_ are shown in Figure 8. The initial state (IS) corresponds to the adsorbed CO_2_. CO_2_ dissociates to form CO* and O*, with CO* desorbing from the surface to ultimately form gaseous CO as the final state (FS). CO_2_ adsorption on Fe sites is exothermic. Two possible reaction pathways for CO_2_ splitting were identified. Both pathways are exothermic (−0.89 eV (−85.87 kJ/mol) and −1.76 eV (−169.82 kJ/mol)), with activation energies of 1.09 eV (−105.17 kJ/mol) and 1.82 eV (−175.60 kJ/mol), respectively. Thermodynamically, both paths are feasible, but Pathway 1 (lower activation energy) is kinetically favored and identified as the optimal pathway.

**Figure 9:**
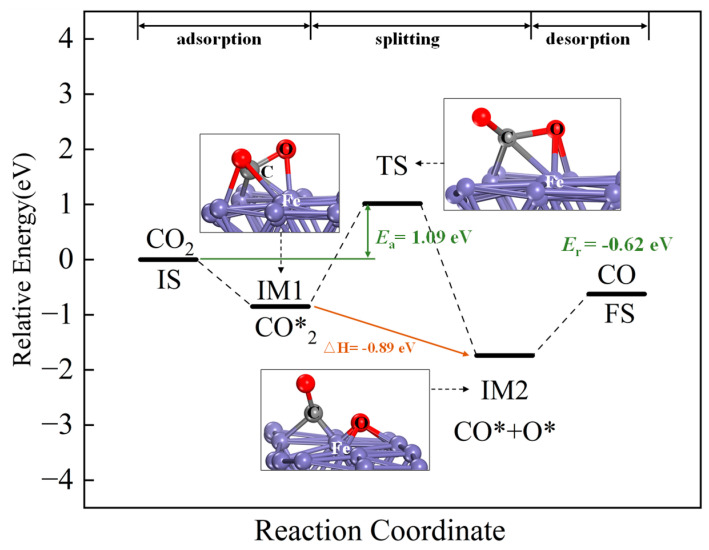
The energy profile of the optimal pathway’s intermediates, transition states and final states of CO_2_ splitting on CaO(111)/Fe(110) surface.

The energy profile of the optimal pathway, including intermediates, transition states and final states, is shown in Figure 9. The dissociation reaction produces CO* and O* adsorbed on the surface. The O atom adsorbs on an Fe site, forming an Fe-O bond (1.9332 Å), close to the Fe-O bond length in Fe_2_O_3_ (1.9440 Å) [41]. The reaction energy is −0.62 eV (−59.82 kJ/mol), corresponding to Fe oxidation. CO desorbs into the gas phase, achieving CO reduction and the oxidative regeneration of reduced Ca_2_Fe_2_O_5_.

### 2.4. H_2_O Adsorption and Splitting

**Figure 10:**
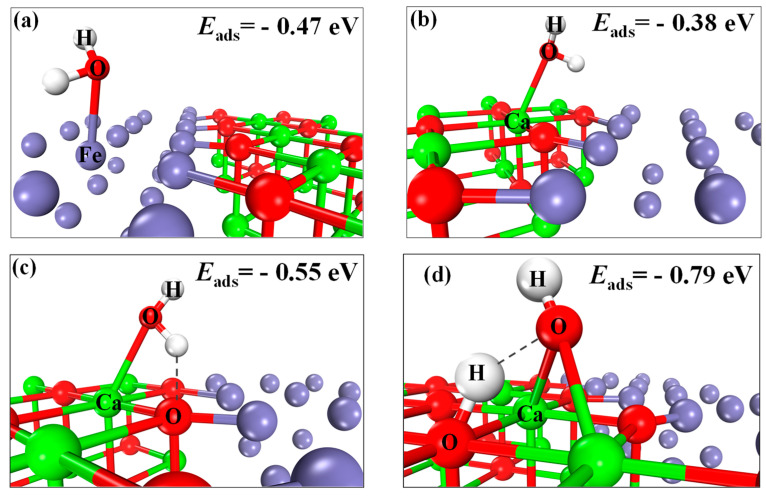
Adsorption configurations of H_2_O on the CaO(111)/Fe(110) surface: (**a**) Fe-top; (**b**) Ca-top; (**c**) Ca-O hole; (**d**) Ca-O bridge.

The adsorption configurations of H_2_O at the CaO(111)/Fe(110) composite interface are shown in Figure 10a–d. Configuration (a) represents H_2_O adsorbed at the Fe site, (b) at the Ca top site, (c) at the Ca hollow site and (d) at the Ca bridge site. The most negative adsorption energy corresponds to the most stable configuration.

When H_2_O adsorbs at the Fe site (Figure 10a), the adsorption energy is −0.47 eV (−71.40 kJ/mol). The most substantial adsorption occurs at the Ca bridge site (Figure 10d), with an energy of −0.79 eV (−76.22 kJ/mol). Configurations (b) and (c) exhibit adsorption energies of −0.38 eV (−36.66 kJ/mol) and −0.55 eV (−53.07 kJ/mol), respectively. Thermodynamic simulations (Figure 5) indicate that CaO and H_2_O cannot spontaneously form Ca(OH)_2_ above 550 °C. However, the reaction between Fe atoms on CaO(111)/Fe(110) and H_2_O has a △G < 0, indicating that the reaction can proceed spontaneously. Thus, this study focuses on Fe sites as the primary adsorption sites. Figure 10a shows the most stable H_2_O adsorption configuration, where the H-O1 and H-O2 bond lengths (0.9792 Å and 0.9805 Å) are 0.0065 Å and 0.0078 Å longer than that of gaseous H_2_O (0.9727 Å), indicating molecular activation. Previous studies reported Fe-site adsorption energies of −0.38 eV [42] and −0.39 eV [43], while the composite interface shows enhanced adsorption (−0.47 eV (−71.40 kJ/mol)), demonstrating its superiority for H_2_O adsorption.

**Figure 11:**
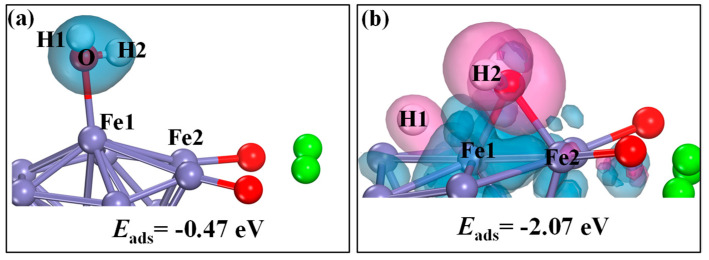
Optimized structures and deformation charge density of H_2_O adsorption over CaO(111)/Fe(110) surface: (**a**) configuration a; (**b**) configuration b.

The optimized structures and deformation charge density of the H_2_O adsorption configurations on the CaO(111)/Fe(110) surface were analyzed, as shown in Figure 11. Figure 11a represents the most stable configuration (a) from Figure 10, where H_2_O undergoes chemisorption atop an Fe atom, forming an Fe-O bond (2.2187 Å) with an adsorption energy of −0.47 eV (−71.40 kJ/mol). The deformation charge density in Figure 11a reveals no significant electron gain/loss in the adsorbed H_2_O molecule before and after adsorption. Specific Bader analysis results (Table 3) indicate electron transfer between H_2_O and Fe. During adsorption, H1, H2 and O act as electron acceptors, while Fe1 and Fe2 are electron donors. The adsorbed H_2_O carries a charge of −0.612 |e|. Configuration (b) in Figure 11 represents the formation of OH* through H atom migration after electron acquisition. The Bader analysis shows H1, H2, Fe1 and Fe2 as electron donors, with O as the acceptor. The total charges of Fe1 before and after adsorption are 0.019 |e| and 0.927 |e|, respectively, confirming Fe1 as an electron donor. This indicates that reduced-state Ca_2_Fe_2_O_5_ acts as the electron donor during the cleavage reaction, while H* serves as the acceptor. The reduced Ca_2_Fe_2_O_5_ is oxidized by H_2_O at high temperatures.

**Figure 12:**
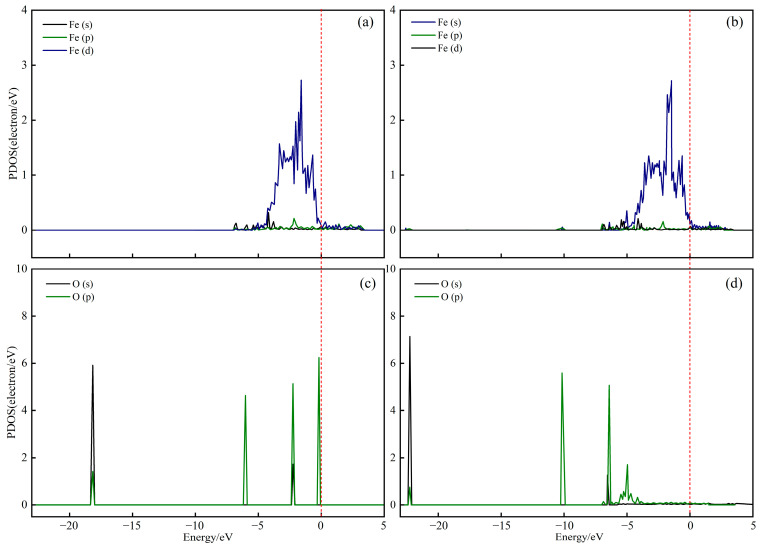
PDOS of H_2_O adsorption on CaO(111)/Fe(110)surface: (**a**,**c**) before adsorption; (**b**,**d**) after adsorption.

The PDOS of Fe atoms and H atoms in OH* on CaO(111)/Fe(110) before and after H_2_O molecule adsorption were calculated. By comparing the PDOS in Figure 12 before and after adsorption, electronic resonances between O and Fe atoms occurred at energies of −22.27 eV and −10.19 eV, indicating the formation of covalent bonds between Fe and O atoms. From the comparison of Figure 12c,d, the P orbitals of the O atoms shifted to lower energy levels, demonstrating that the adsorption of H_2_O on the Fe atoms of CaO(111)/Fe(110) becomes more stable through the formation of new molecular orbitals.

**Figure 13:**
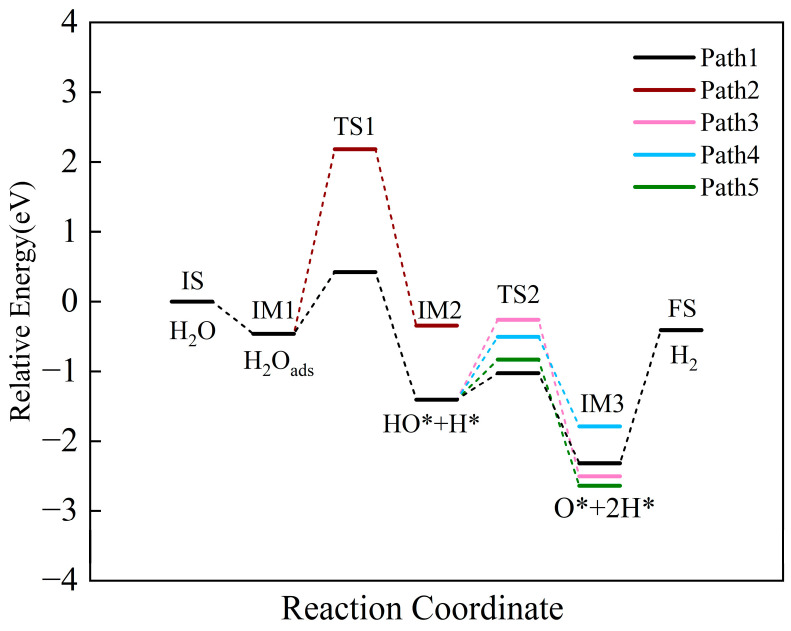
The energy profile of intermediates, transition states and final states of H_2_O splitting on the CaO(111)/Fe (110) surface.

After H_2_O is adsorbed on the CaO(111)/Fe(110) surface, the H_2_O undergoes a dissociation reaction. From the above analysis, H_2_O can be chemically adsorbed stably on the composite surface as the initial state (IS) for the H_2_O reduction process. Figure 13 shows the reaction pathway and energy profile for H_2_O splitting on the composite surface and the oxidation of the reduced Ca_2_Fe_2_O_5_ state.

At the transition state TS1, there are two reaction pathways. The activation energies for Pathway 1 and Pathway 2 are 0.42 eV (40.52 kJ/mol) and 2.18 eV (210.34 kJ/mol), respectively. Pathway 1 is exothermic, with heat release of −0.94 eV (−90.70 kJ/mol), while Pathway 2 is endothermic, with heat absorption of 0.02 eV (1.93 kJ/mol). Therefore, Pathway 1 is the optimal reaction path. The initial dissociation of H_2_O forms HO* and H*.

**Figure 14:**
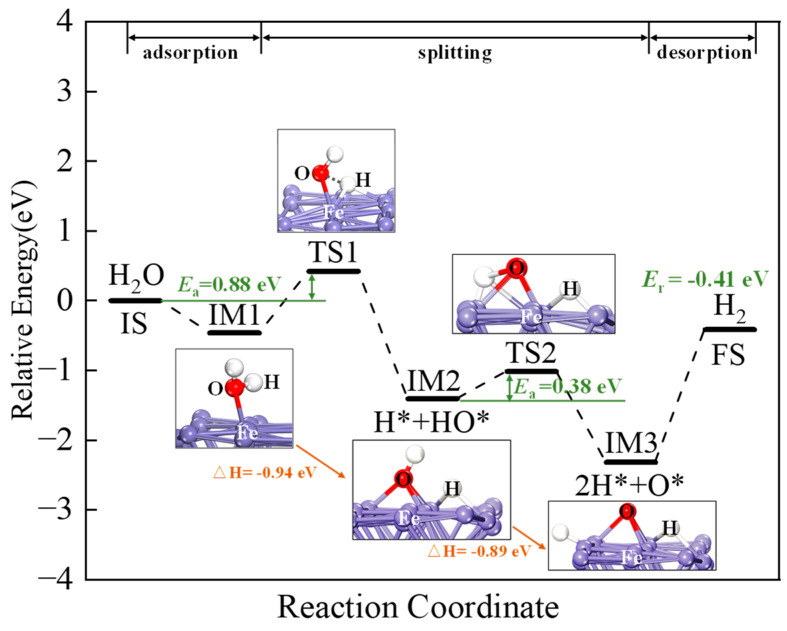
The energy profile of the optimal pathway’s intermediates, transition states and final states of H_2_O splitting on CaO(111)/Fe(110) surface.

At transition state TS2, there are a total of four reaction paths. The adsorbed HO* undergoes further dissociation to form H* and O*. When HO* is adsorbed on the surface, four different adsorption sites exist, resulting in four distinct reaction pathways: Paths 1, 3, 4, and 5. The heat release values are −0.91, −1.23, −1.10, and −0.65 eV (−87.80,−118.68,−106.13, and −62.72 kJ/mol), respectively, all being exothermic reactions. Therefore, all pathways are thermodynamically favorable. The activation energies are 0.38, 1.15, 0.90, and 0.58 eV (36.66, 110.96, 86.84, and 55.96 kJ/mol), respectively. Path 1 has the lowest activation energy, making it the most favorable pathway. The reaction is most likely to proceed through Path 1, which can thus be considered the optimal reaction pathway. The activation energies for transition states TS1 and TS2 are 0.42 eV (40.52 kJ/mol) and 0.38 eV (36.66 kJ/mol), respectively. Since TS1 has higher activation energy than TS2, TS1 determines the dissociation rate of H_2_O on the composite surface. H_2_O dissociates into two H* and one O* on the composite surface. The O* adsorbs on Fe sites, forming Fe-O bonds with surface Fe atoms. The two H* combine to form adsorbed H_2_, which desorbs from the surface to form gaseous H_2_. As shown in Figure 14, the overall reaction energy is −0.41 eV (−39.56 kJ/mol), indicating that the H_2_O reduction to form H_2_ is an exothermic reaction.

## 3. Materials and Methods

### 3.1. Material Preparation

The *Chlorella vulgaris* (biomass) was provided by Xi’an Shengqing Biotechnology Co., Ltd. (Xi’an, China). First, the biomass was dried at 105 °C for 24 h. It was then sieved into particles with a size of less than 100 μm and stored in a sealed desiccator. The ultimate and proximate analyses of the biomass are shown in Table 4.

The Ca_2_Fe_2_O_5_ oxygen carrier was prepared via the sol–gel method. Ca(NO_3_)_2_·4H_2_O (analytical reagent), Fe(NO_3_)_3_·9H_2_O (analytical reagent) and citric acid were dissolved in deionized water at a molar ratio of 1:1:2. The solution was stirred until a viscous substance formed. The obtained viscous material was dried at 105 °C for 24 h and then calcined in a muffle furnace at 900 °C for 4 h. Finally, the solid was ground and sieved to below 100 μm for characterization. SiO_2_ (analytical reagent) was used as a control in the experiments.

### 3.2. Material Characterization

The phase composition of the oxygen carrier was determined using an X-ray diffractometer (XRD). A Rigaku Ultima IV X-ray diffractometer (Tokyo, Japan) was employed, with the experimental conditions consisting of a Cu target, a scanning rate of 2°/min and a collection range of 10~90°.

To verify the temperature-programmed reduction (TPR) performance of the oxygen carrier, hydrogen temperature-programmed reduction (H_2_-TPR) experiments were conducted under a hydrogen reduction atmosphere using an Micromeritics Auto Chem II 2920 instrument (Norcross, GA, USA). Temperature-programmed analysis is a dynamic process that detects changes in surface chemical properties under different atmospheres at specific heating rates under inert gas protection. Typically, 50 mg of oxygen carrier was pretreated by heating to 150 °C under an inert atmosphere and maintained for 30 min. After pretreatment, the temperature was programmed to cool to 30 °C. Once the instrument baseline stabilized, the oxygen carrier was heated to 900 °C at 10 °C/min under a mixed atmosphere of H_2_ (10 vol.%)/N_2_ (50 mL/min flow rate).

### 3.3. Chemical Looping Gasification Experiment

**Figure 15:**
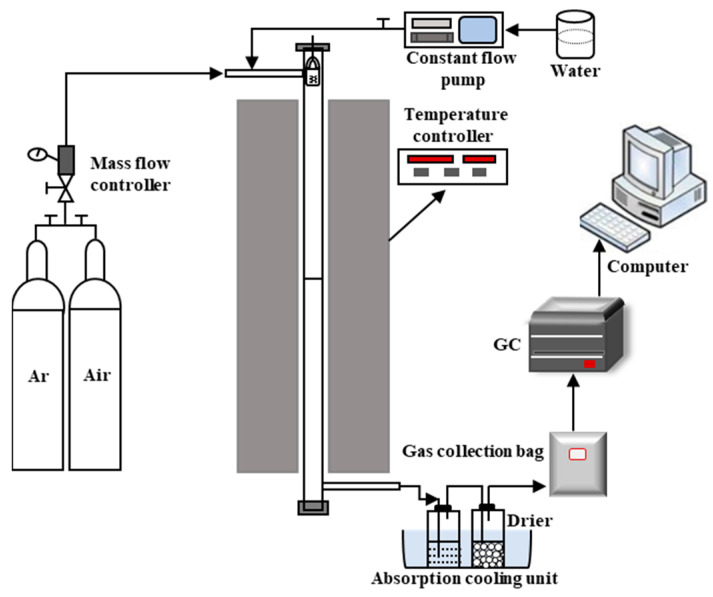
Schematic of fixed bed experiment.

Figure 15 shows the fixed-bed setup for the gasification experiments. It mainly consisted of a gas supply system, reaction system, absorption–condensation–drying system, gas collection system and detection system. Argon was used as the carrier gas to maintain the required inert atmosphere during gasification. Steam was continuously fed into the reactor via a constant-flow pump.

Before each experiment, the biomass and oxygen carrier were uniformly mixed at a specific mass ratio and loaded into a basket suspended in the upper part of the tubular furnace. The system was then sealed. When the furnace reached 800 °C, the basket was pulled to drop into the constant-temperature zone for reaction. In the blank experiments, SiO_2_ replaced the oxygen carrier. Argon was purged into the furnace at 100 mL/min. Deionized water was pumped into the tubular furnace using the constant-flow pump, where it rapidly vaporized and was carried by the argon into the reaction zone. The reaction duration was set to 30 min. The generated gases were collected in gas bags and analyzed using gas chromatography.

### 3.4. Gasification Performance Evaluation Indicators

As the content of C_2_ and alkanes and olefins was relatively low, the main gases studied included the following. The definitions and calculation formulas for the chemical looping gasification evaluation indicators are shown below.

The gas yield *G*_v_ (Nm^3^/kg) is calculated via Equation (1):

(1) $$G_{V}=V_{g}/m_{B}$$

where *V*_g_ represents the volume of the produced gas under standard conditions (273.15 K, 101,325 Pa), Nm^3^; *m*_B_ represents the mass of biomass used in the gasification process, kg.

The lower heating value of the gas (*LHV*) (kJ/Nm^3^) is calculated via Equation (2):

(2) $$LHV=126\cdot V_{\mathrm{CO}}+108\cdot V_{H_{2}}+359\cdot V_{{\mathrm{CH}}_{4}}+635\cdot V_{C_{2}H_{m}}$$

$V_{\mathrm{CO}}$, $V_{H_{2}}$, $V_{{\mathrm{CH}}_{4}}$ and $V_{C_{2}H_{m}}$ represent the volume fractions (%) of CO, H_2_, CH_4_ and C_2_H_m_.

The carbon conversion efficiency *η*_c_ (%) is calculated via Equation (3):

(3) $$\eta_{c}=\frac{12\times \left(V_{CO}+V_{CO_{2}}+V_{CH_{4}}+2V_{C_{2}H_{m}}\right)\times G_{v}}{22.4\times \left(T_{1}/T\right)\times C\%}$$

where *T*_1_ represents the temperature (K) during gas concentration measurement; *T* represents the standard temperature (K); *C* % represents the carbon content in the biomass.

The gasification efficiency *η* (%) is calculated via Equation (4):

(4) $$\eta =\frac{LHV\times G_{v}}{Q_{B}}$$

where *Q*_B_ represents the lower heating value of the biomass, kJ/Nm^3^.

The selectivity of CO is given by Equation (5):

(5) $$CO\;selectivity=\frac{G_{CO}}{G_{CO}+G_{CO_{2}}}\times 100\%$$

### 3.5. Computational Details

**Figure 16:**
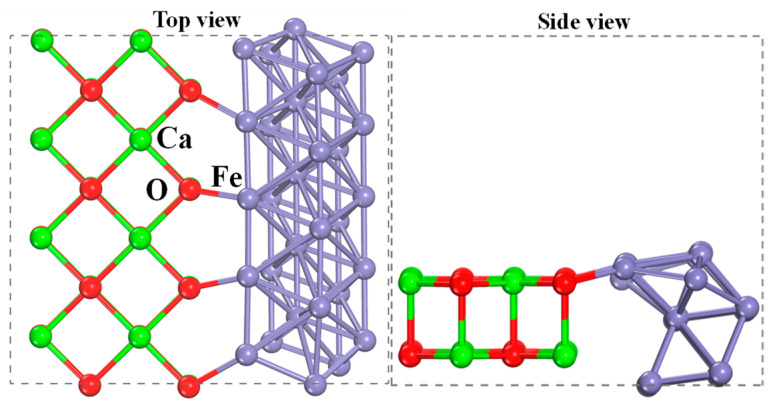
Views of the composite CaO(111)/Fe(110) configuration.

All calculations were implemented in CASTEP of Materials Studio 6.0. The exchange-correlation energy was treated using the Perdew–Burke–Ernzerhof generalized gradient approximation (GGA-PBE) and the projector augmented wave (PAW) method [36,37,38,39,40]. Dispersion corrections and spin polarization effects were considered. The plane-wave cutoff energy was set to 400 eV. The CaO(111)/Fe(110) surface was constructed to simulate the reduced Ca_2_Fe_2_O_5_ (as shown in Figure 16). The k-point sampling of the Brillouin zone was set to 1 × 1 × 1. A vacuum layer of 15 Å was introduced. Similarly, the molecular structures of CO_2_, CO, H_2_O and H_2_ were optimized.

The fully linear synchronous transit (LST)/quadratic synchronous transit (QST) [44] search protocol was employed to establish pathways between the reactants and products, locating transition states through interpolated reaction path synchronous transition methods. This approach performs LST calculations and iteratively applies conjugate gradient optimization followed by QST processing until the simulation steps terminate. Multiple path optimizations were executed until achieving transition state structures, avoiding unrealistic reaction paths caused by speculative intermediates.

Surface adsorption energy *E*_ads_:

*E*_ads_ = *E*_total_ − *E*_surface_ − *E*_species_ (6)

*E*_total_ represents the total energy of the adsorbed surface species; *E*_surface_ denotes the energy of the clean surface without adsorption; *E*_species_ is the energy of the substance in the gas phase.

Activation energy *E*_a_ and reaction energy *E*_r_:

*E*_a_ = *E*_TS_ − *E*_IS_ (7)

*E*_r_ = *E*_FS_ − *E*_IS_ (8)

*E*_TS_ is the energy of the transition state; *E*_IS_ is the energy of the initial state; and *E*_FS_ is the energy of the final state.

This study did not incorporate zero-point energy (ZPE) corrections, following the general approach commonly used in the dependency analysis of similar periodic systems. The calculated adsorption energy values are consistent with those reported in reference [38].

## 4. Conclusions

This study reveals the multiscale mechanism of Ca_2_Fe_2_O_5_ oxygen carriers in biomass direct/steam chemical looping gasification. The results demonstrate that introducing an oxygen carrier significantly enhances the gasification performance, with the CO yield doubling (increasing from 0.13 Nm^3^/kg to 0.26 Nm^3^/kg). The H_2_ yield increases from 0.19 Nm^3^/kg to 0.72 Nm^3^/kg. This indicates that reduced Ca_2_Fe_2_O_5_ can regenerate to its initial state through oxidation by CO_2_/H_2_O at high temperatures, accompanied by efficient CO/H_2_ release. Combined with DFT calculations, the reaction mechanisms of CO_2_/H_2_O at the active interface of the reduced oxygen carrier are systematically elucidated. CO_2_/H_2_O are stabilized via chemisorption at the CaO(111)/Fe(110) composite interface. Adsorbed CO_2_ (total charge −0.952 |e|) and H_2_O (total charge −0.612 |e|) act as electron acceptors, while surface Fe atoms (charges 0.433 |e|, 0.927 |e|) serve as electron donors, driving efficient reactant activation. CO_2_ undergoes single-step splitting (CO_2_→CO* + O*) to form adsorbed CO, with its desorption energy barrier (*E*_a_ = 1.09 eV, 105.17 kJ/mol) determining the reaction rate. H_2_O splits in two steps (H_2_O→HO* + H*→2H* + O*), with the first step dictating the reaction rate (*E*_a_ = 0.42 eV, 40.52 kJ/mol). Overall, CO_2_/H_2_O enable oxidative regeneration through surface reactions on reduced Ca_2_Fe_2_O_5_ while supplying heat to the system. This work establishes a theoretical framework for the precision design of high-performance oxygen carriers and opens up new pathways for the selective regulation of biomass gasification products.

## Figures and Tables

**Table 1:** Design of chemical looping gasification experiments.

	Biomass	Oxygen Carrier	Steam (mL/min)	Temperature (°C)	**Time (min)**
A1	*Chlorella vulgaris*	SiO_2_	/	800	30
A2	*Chlorella vulgaris*	Ca_2_Fe_2_O_5_	/	800	30
A3	*Chlorella vulgaris*	Ca_2_Fe_2_O_5_	0.04	800	30

**Table 2:** Bader charge analysis.

Configuration		C	O1	O2	Fe1O	Fe2C
a	Before adsorption	1.626	−0.812	−0.814	−0.057	0.172
	After adsorption	0.918	−0.907	−0.963	−0.109	0.503
b	Before adsorption	1.236	−1.087	−0.814	−0.057	0.172
	After adsorption	0.421	−0.974	−0.868	−0.605	0.433

**Table 3:** Bader charge analysis.

Configuration		H1	H2	O	Fe1	Fe2
a	Before adsorption	0.581	0.551	−1.132	0.019	−0.011
	After adsorption	0.329	0.231	−1.172	0.198	0.391
b	Before adsorption	0.513	0.551	−1.132	0.018	−0.011
	After adsorption	0.431	−0.467	−0.871	0.927	0.198

**Table 4:** Proximate and ultimate analyses of biomass.

Proximate Analysis (wt.%, ad)				Ultimate Analysis (wt.%, ad)					Low Heating Value (MJ/kg)
Moisture	Volatiles	FC	Ash	C	H	N	S	O ^a^	
4.40	76.63	14.55	6.42	48.66	6.96	9.26	0.65	34.47	19.23

^a^ Difference calculation.

## Data Availability

The original contributions presented in this study are included in the article. Further inquiries can be directed to the corresponding author.
